# Extracellular Vesicles and PD-L1—A Review of Complex Immunoregulatory Properties and Clinical Importance

**DOI:** 10.3390/biomedicines13061356

**Published:** 2025-05-31

**Authors:** Kajetan Kiełbowski, Paulina Plewa, Jacek Szulc, Maciej Ćmil, Estera Bakinowska, Andrzej Pawlik

**Affiliations:** Department of Physiology, Pomeranian Medical University, 70-111 Szczecin, Poland; paulina.plewa@op.pl (P.P.); esterabakinowska@gmail.com (E.B.)

**Keywords:** extracellular vesicles, cancer, autoimmune diseases, programmed cell death protein 1

## Abstract

Extracellular vesicles (EVs) are membrane-bound structures released by cells that contain bioactive cargo such as cytokines or non-coding RNA. It is widely known that EVs influence the activity of other cells; they take part in the pathogenesis and compensatory mechanisms of multiple diseases. Frequently, EVs can share the properties of their source cells, thus allowing the use of EVs as non-cellular vectors or therapeutic agents. Importantly, these structures can express the ligand for the programmed cell death protein 1 (PD-L1). It binds to the PD-1 protein present on the immune cells, which suppresses the activity of T cells. The PD-1/PD-L1 axis is widely known in the field of oncology, as PD-L1 present on the surface of cancer cells inhibits cytotoxic activity of T cells, thus promoting cancer growth and treatment resistance. Immunotherapy prevents PD-1/PD-L1 binding and restores anticancer properties of the immune cells. By contrast, the above-mentioned binding is desired in the context of autoimmunity, where abnormal activity of immune cells is a hallmark element in the pathogenesis of these conditions. The aim of this review is to present and discuss the latest findings regarding the role of EVs-PD-L1 in cancer and autoimmunity.

## 1. Introduction

Extracellular vesicles (EVs) are a diverse group of membrane vesicles which contain bioactive cargo and regulate cellular behavior [1,2,3,4]. They are present in many supernatants of biological fluids, e.g., blood, saliva, urine, breast milk, bronchoalveolar lavage fluid, synovial fluid and amniotic fluid [3,5]. Several EV subtypes can be distinguished—they can differ in size, function, biophysical properties, and biogenesis pathways [1]. Apoptotic bodies are released during programmed cell death and their dimensions range from 500 to 2000 nm. Microvesicles (100–1000 nm) are formed as a result of budding of the plasma membrane [5]. In the case of exosomes (40–150 nm), their formation process is related to the endocytic pathway. Late endosomes, also referred to as multi-vesicular bodies (MVBs) fuse with the plasma membrane and release intravesicular bodies into the extracellular space [4,5,6]. Oncosomes (1–10 μm) are released by cancer cells and contain oncogenic material [6,7]. Exophers, with a size of nearly 4 μm, contain mostly organelles and protein aggregates, while migrasomes, whose size does not exceed 1 μm, mainly depend on cell migration within the alveolar secretion mechanism [8]. The coronal layer on the surface of EVs consists of nucleic acids, proteins, lipids, and glycans. The main tasks of the molecules on the surface of EVs are to initiate integration with cells and the microenvironment [9,10].

Macrophages, T and B lymphocytes and natural killer cells (NK cells) release significant amounts of EVs. These structures have immunoregulatory properties and can resemble the functionality of their source cell, thus influencing immune homeostasis [10,11]. For instance, the properties of EVs released from macrophages depend on the pro- or anti-inflammatory properties of their source cell. These vesicles affect polarization of macrophages [10,12], including those present in the tumor microenvironment (TME) [13], thus affecting cancer progression and treatment response.

The bioactive immunoregulatory cargo of EVs is composed of molecules that regulate cellular behavior, such as cytokines and non-coding RNA. Programmed cell death ligand 1 (PD-L1) is also expressed in EVs, which highlights important immunoregulatory features of these structures [10,14]. PD-1 is an immune checkpoint found on the surface of immune cells, such as T and B cells, NK cells, monocytes, and some DCs. The ligands of PD-1 are PD-L1 and PD-L2. Interaction of receptors with ligands causes stimulation of intracellular signaling pathways that inhibits the stimulation of immune cells. As a result, immune cells secrete less cytokines which prevents the occurrence of a phenomenon known as immune exhaustion.

Under physiological conditions, the PD-1/PD-L1 pathway is responsible for inducing appropriate physiological effects to maintain adequate immune homeostasis [15]. The PD-1/PD-L1 interaction is primarily associated with suppression of T cells. The axis precisely regulates the stimulation mediated by the T cell receptor (TCR). Specifically, it inhibits the expression of genes mediating survival and apoptosis of T cells. Consequently, it reduces the possibility of cytokine release by these cells. In B cells, stimulation is inhibited by the B cells receptor (BCR). In addition, PD-1 can suppress the immune response of the described cells to antigens [16].

Weakening of the PD-1/PD-L1 axis significantly increases the predisposition to autoimmune diseases, including rheumatoid arthritis (RA), multiple sclerosis and systemic lupus erythematosus (SLE). These diseases are mainly associated with the polymorphism of the PDCD1 gene, which encodes PD-1. In the pathogenesis of RA, the PD-1/PD-L1 pathway is postulated to be a protective element [17,18]. It may be related to the inhibition of Th17 cell differentiation as a result of inhibitory action on the PI3K/Akt/PKCθ pathway [19]. However, it was also shown that PD-1 expression on CD4+ and TCD8+ T cells is inversely correlated with DAS (Disease Activity Score) [20]. In RA, synovial fluid and peripheral blood can demonstrate increased levels of T cells, along with PD-1, which are expressed on their surface [21]. PD-1 deficiency results in excessive proliferation of T cells with dysregulated Th17 cell responses and increased release of IFN-γ and IL-17 [19]. SLE is associated with the stimulation of self-reactive T and B cells, but also with tissue defects caused by the presence of deposits of the immune complex leading to inflammation and, consequently, failure of many organs. In this disease, PD-1 has been found to adversely affect the regulation of T cell stimulation, but it is necessary to maintain normal Treg function, thus controlling the autoimmunity [18,22]. In addition, many patients have been found to have reduced PD-1 expression, which may suggest an essential role for this protein in the pathogenesis of SLE [23].

**Figure 1:**
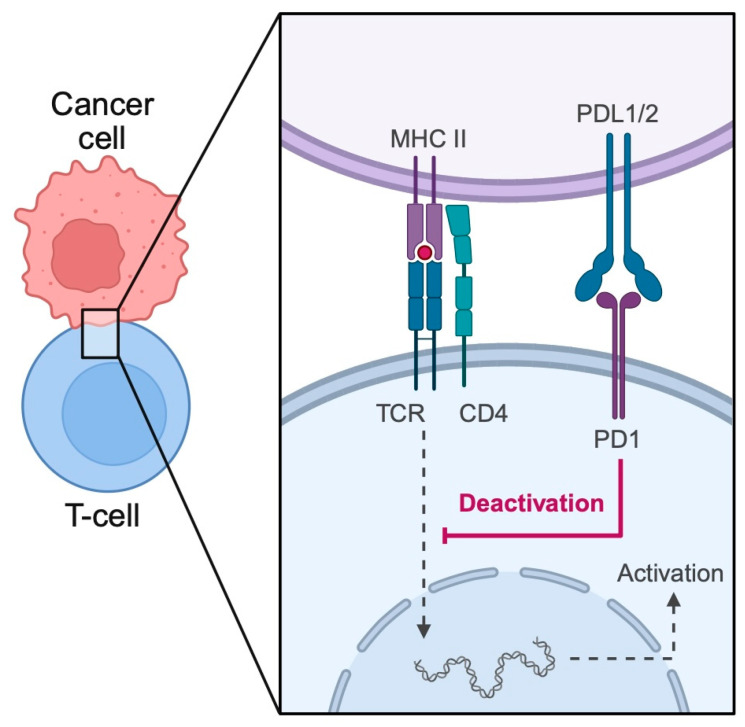
The role of PD-1/PD-L1 binding in immune evasion by cancer cells. PD-L1 expressed on malignant cells binds to PD-1 present on T cells, which suppresses cytotoxic properties of the immune system. Created in BioRender. Physiology, D. (2025) https://BioRender.com/dp8i62e.

Importantly, PD-1/PD-L1 plays a crucial role in cancer. Tumor cells express PD-L1 that can suppress the activity of cytotoxic T cells by binding to the PD-1 expressed on the surface of immune cells (Figure 1). As a result, immunotherapy in the form of immune checkpoint inhibitors (ICIs) has been widely introduced in the clinical practice in the field of oncology. Drugs such as pembrolizumab, nivolumab, and durvalumab represent some of the ICIs used in the treatment of cancer. These therapeutics reverse T cell suppression, thus allowing the immune system to target and inhibit cancer progression.

The aim of this review is to discuss the latest findings on the involvement of EVs expressing PD-L1 in autoimmune diseases and cancer. We will present the involvement of these structures in the pathogenesis of diseases and their potential targeting in the treatment.

## 2. Extracellular Vesicles and PD-1—The Relationship with Pathological Conditions

### 2.1. Cancer

Cancer cells are characterized by a number of properties that promote tumor growth. These features include altered cellular metabolism, proliferation stimulation, and resistance to apoptosis. Furthermore, malignant cells demonstrate immunoregulatory mechanisms that suppress the anticancer activity of immune system [24,25]. Tumors create a microenvironment that supports their survival. Specifically, cancers influence the composition of a specific niche known as the TME. It is a complex ecosystem consisting of tumor cells, immune cells, blood vessels, and signaling molecules that can either promote or inhibit tumor growth. It is infiltrated by several immunosuppressive cells, including T regulatory cells (Tregs), tumor-associated macrophages (TAMs) and myeloid-derived suppressor cells (MDSCs). Additionally, both tumor and immune cells in the TME exhibit elevated expression of critical immune checkpoints, such as CTLA-4, PD-1 and its ligand PD-L1. Tumors secrete various factors—some of which are not yet fully understood—that influence the behavior of certain immune cells, such as macrophages and lymphocytes. These alterations likely promote angiogenesis, tissue fibrosis, and the accumulation of M2 macrophages. Instead of mounting a protective anti-tumor immune response, these changes may instead support tumor growth [26,27,28,29,30,31].

**Figure 2:**
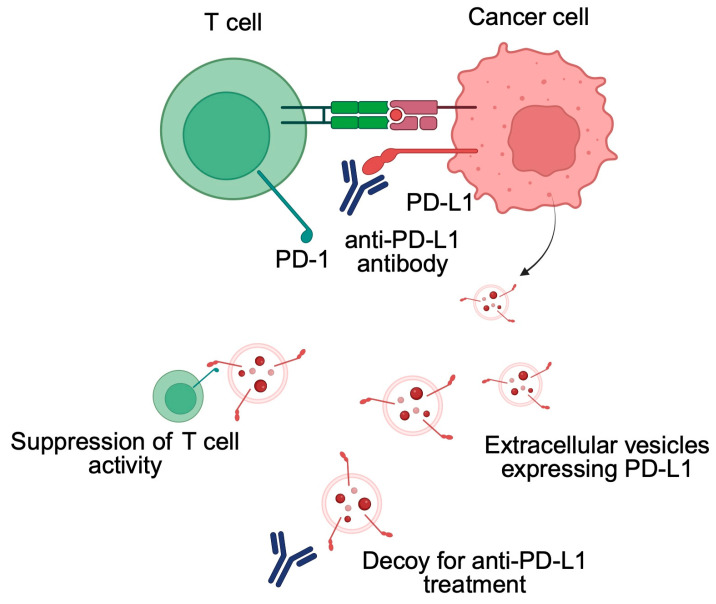
Cancer cells secrete extracellular vesicles with PD-L1 that can suppress the activity of T cells and promote treatment resistance. Created in BioRender. Physiology, D. (2025) https://BioRender.com/ee43lc3.

Investigations have revealed that EVs-PD-L1, secreted by cancer cells, interact with PD-1 on CD8+ T cells, leading to T cell inactivation and contributing to tumor immune evasion. This interaction impairs the proliferation and function of effector T cells, resulting in reduced expression of activation markers like CD69 and decreased secretion of cytokines such as IFN-γ, TNF-α, and IL-2, thus contributing to immune suppression and tumor progression. Moreover, EVs secreted by cancer cells affect the behavior of immune cells present in TAM to modulate their expression of PD-L1. For example, Yin et al. demonstrated that CRC cells can promote the expression of PD-L1 on the surface of TAMs through EVs [32]. EVs expressing PD-L1 are involved in mediating other crucial aspects in cancer immunotherapy—the treatment resistance. As is widely known, not all patients will respond to immunotherapy. Furthermore, acquired resistance can develop after the initial response to treatment. Researchers are actively searching for mechanisms responsible for treatment resistance to improve clinical benefits. EVs with PD-L1 can decoy anti PD-L1 antibodies, thus limiting their interaction with ligands expressed on the cancer cells [33] (Figure 2).

Recent studies suggest that tumor-derived exosomes (TDEs) carry immunosuppressive signals that inhibit the proliferation and function of T cells within the TME. The Fas/FasL signaling pathway is a key regulator of T cell apoptosis, and TDEs have been shown to express membrane-bound FasL, selectively inducing T cell apoptosis or suppressing TCR signaling by reducing the expression of CD3-ζ. TDEs have also been found to stimulate the generation and proliferation of Tregs. In colorectal cancer, TDEs enriched with TGF-β activate the TGF-β/Smad signaling pathway, leading to the upregulation of genes associated with Tregs.

B cells also play essential roles in the immune response, including producing immunoglobulins, presenting antigens, delivering costimulatory signals, and secreting cytokines to regulate antitumor immunity. A subset of B cells, known as Bregs, can suppress adaptive immunity by secreting inhibitory cytokines such as IL-10, IL-21, IL-35, and TGF-β1, or by expressing PD-L1. The generation of Tregs and the activity of MDSCs and CD4+ T cells are also regulated by Bregs. For example, exosomes from esophageal cancer cells promoted the differentiation of naïve B cells into TGF-β-producing Bregs, which inhibited the proliferation of CD8+ T cells through immunosuppressive effects. Additionally, TDEs carrying high mobility group protein B1 (HMGB1) have been reported to trigger the differentiation of TIM-1+ Bregs, which suppress the cytotoxic activity of CD8+ T cells and support tumor survival and metastasis [34,35,36,37,38].

Anti-PD-1 and PD-L1 antibodies restore anti-tumor immune responses. This is reflected by an increase in the number of T cells and B cells in the TME during treatment with PD-1 inhibitors. In responders, a subset of CD8+ T effector memory cells is particularly abundant in the tumor [39]. FDA-approved anti-PD-1 and PD-L1 antibodies with cancer types for which they are approved are listed in Table 1.

Therapies targeting PD-1/PD-L1 axis have different success rates depending on the type of malignancy and treatment strategy. There is a need to stratify patients who will benefit the most from treatment [50]. One of the methods to do this is based on the expression of PD-L1 in tumor samples assessed by immunohistochemical staining. This method has an AUC of 0.60 (95% CI, 0.55–0.64) irrespective of cancer type [51]. In some neoplasms, the combined positive score (CPS) of PD-L1 expression determines the introduction of immunotherapeutics. The PD-1/PD-L1 inhibitors are frequently combined with chemotherapy. In a large meta-analysis, the objective response rate (ORR) for ICI + chemotherapy combination was 46.81% [95% CI: 36.02–57.60] in comparison with 17.75% [95% CI: 14.47–21.03] for ICI monotherapy [50]. The aforementioned promotion of immune responses by ICIs may result in a broad spectrum of auto-immune toxicities called immune-related adverse events [52]. Nevertheless, the precise treatment strategies vary significantly depending on the cancer type. For instance, a combination of two immunotherapeutics is clinically used in the treatment of melanoma (nivolumab + ipilimumab). Exosomes expressing PD-L1 were proven to contribute to cancer development and clinical outcomes. Firstly, high levels of exosomal PD-L1 were correlated with poorer outcomes of patients with gastric cancer. Mechanistically, these structures were associated with increased presence of MDSCs in the TME. Interestingly, EVs could induce the proliferation of MDSCs through the IL-6/STAT pathway [53]. Thus, PD-L1 exosomes promote immunosuppressive conditions in the TME not only through the suppression of T cell activity, but also through the expansion of immunosuppressive cells. As previously mentioned, features of EVs frequently resemble those of their origin cells. Recently Sanchez et al. [54] demonstrated that specific subtype of glioma cells secretes EVs with PD-L1, thus suppressing the activation of T cells. Specifically, researchers showed that vesicles are secreted by malignant cells that lost phosphatase and tensin homolog (PTEN), one of the primary tumor suppressors in the PI3K/Akt signaling cascade. Similar findings were recently described regarding epithelial growth factor receptor (EGFR) mutated NSCLC [55]. Perhaps, we could monitor EVs to analyze aberrations in signaling pathways and understand molecular basics of oncology in individual patients. Hypothetically, monitoring of PD-L1 (+) EVs could be utilized in the future to identify potential biomarkers of response or treatment targets. Monitoring of these structures in peripheral blood would represent an innovative method of liquid biopsy.

It is a minimally invasive technique in which the collected samples of blood or other body fluids are used to detect circulating tumor cells (CTC), EVs or molecules such as miRNA, lnRNA, circRNA, and ctDNA [56]. It is considered that liquid biopsy will supplement diagnostics, prognosis assessment, treatment selection, monitoring of treatment response and detection of residual disease [57]. The use of PD-L1 EVs as liquid biopsy markers has been studied in recent years. For example, PD-L1 EVs level in plasma was demonstrated to be of prognostic value for diffuse large B cell lymphoma (DLBCL), colorectal cancer liver metastases, gastric cancer and lung cancer [58,59,60,61]. The use of PD-L1 (+) small EVs concentration in plasma as a prognostic factor in patients with non-small cell lung cancer (NSCLC) was investigated by Eslami-S et al. Increase in PD-L1 (+) small EVs concentration was significantly associated with worse overall survival in NSCLC patients (HR _increase of 5pg/mL_ = 1.14, 95% CI = 1.03–1.26, *p* = 0.016). Moreover, the combination of PD-L1 (+) small EVs with other prognostic markers was investigated. The hazard ratio for overall survival in patients with combination of high concentration of PD-L1 (+) small EVs, presence of CTC, presence of ctDNA mutations was 8.88, 95% CI (2.79–28.31), *p* < 0.001 [61]. In addition, exosomal PD-L1 blood concentration was shown to correlate with nodal metastasis in lung cancer [62].

Currently it is recommended to qualify patients for ICI therapy in NSCLC based on detection of PD-L1 in tumor samples with the use of IHC [63]. PD-L1 IHC has both sensitivity and specificity of 63% in identifying patients that benefit from ICI therapy in NSCLC [51]. Moderate predictive value of PD-L1 IHC justifies the investigation of new liquid biopsy markers for ICI therapy response. Interestingly, it was demonstrated that PD-L1 EVs plasma level correlates with expression of PD-L1 in tumor [64]. In accordance with this finding, the use of PD-L1+ large EVs level in plasma for identifying ICI-responders in NSCLC was investigated. It was demonstrated to have a promising predictive value with an AUC of 0.79 in all patients, irrespective of tissue PD-L1 status. In tissue PD-L1 negative patients the predictive value was even greater with an AUC of 0.91 [65]. Therefore, PD-L1 (+) large EVs level could be potentially used to predict response to ICIs specifically in tissue PD-L1 negative patients. Moreover, dynamics of all type PD-L1 EVs concentration in plasma during ICI therapy differ between responders and non-responders and therefore can be used to predict durable response in NSCLC. PD-L1 EVs level during therapy is increasing in non-responders and decreasing in responders. In a study by de Miguel-Perez et al., the PD-L1 EVs dynamics demonstrated predictive efficacy for durable ICI response with an AUC of 77.3% in pembrolizumab-treated cohort and AUC of 75% in pembrolizumab + docetaxel cohort [66].

Since cancer cells-derived PD-L1 EVs play a role in suppressing the activity of immune cells in tumor and consequently immune evasion, a number of preclinical studies investigated potential therapies based on the inhibition of biogenesis and secretion of PD-L1 EVs by cancer cells [67,68]. To begin with, LAMTOR1—a protein modulating lysosomal trafficking, was shown to diminish exosomal PD-L1 secretion in NSCLC. Mechanistically, LAMTOR1 interacts with HRS—a protein known to enrich exosomes in PD-L1. HRS is a protein associated with endosomal sorting complex required for transport which is essential for recognition of ubiquitinated proteins during exosome biogenesis. Interaction between LAMTOR1 and HRS results in activation of lysosomal degradation of PD-L1. Therapy with the combination of LAMTOR1 peptide and anti-PD-1 agent resulted in prolonged survival in comparison with monotherapy in orthotopic mouse model of lung cancer [69,70,71]. A similar approach was recently published by Wang and collaborators [72]. The authors studied mechanisms leading to PD-L1 (+) EVs release from cancer cells. They found that milk fat globule-epidermal growth factor 8 (MFGE8) activates the processes leading to PD-L1 accumulation in the EVs. Researchers further examined translational potential of the pathway and found that monoclonal antibodies targeting MFGE8 combined with anti-PD-1 therapy in animal cancer models resensitized tumors to PD-1 blockade.

Shin et al. showed that sulfisoxazole—a sulfonamide antibiotic—suppresses exosomal PD-L1 which increases ICI treatment efficacy [73]. Sulfisoxazole exerts its inhibitory effect on endosomes via binding with endothelin receptor A on cell surface which leads to suppression of biogenesis and secretion of exosomes and facilitation of lysosomal degradation [74]. EP16—a moclobemide based compound, is another exosomal PD-L1 inhibitor which can be potentially used as anti-PD-1 therapy sensitizer. In an animal model of gastric cancer, a combination of EP16 and anti-PD-1 agent had a tumor growth inhibitory rate of 69%, whereas monotherapy with EP16 and anti-PD-1 had TGI of 32% and 51%, respectively [75]. An interesting method of exosomal PD-L1 expression inhibition and its application was investigated by Zhu et al. Researchers constructed a tumor specific nanomodulator containing amlodipine—a calcium channel blocker and GW4869—a neutral sphingomyelinase (nSMase) inhibitor [76]. Amlodipine-induced reduction in intracellular calcium concentration inhibits calpain-mediated degradation of Beclin1. This results in autophagy of recycling endosomes which are abundant in PD-L1 [77]. Simultaneously, GW4869 inhibits nSMase which results in reduction in bioactive lipid ceramides synthesis and therefore reduced exosome formation [78]. In hepatocellular carcinoma preclinical models, a dual anti-exosomal PD-L1 therapy was effective in reversing the immunosuppressive TME, thus suppressing metastasis formation and progression [76]. Exosomal PD-L1 was reported to mediate oxaliplatin resistance in an animal model of colorectal cancer, possibly via influence on DNA damage response. Secretion of exosomal PD-L1 in colorectal cancer cells is induced by type Iγ phosphatidylinositol phosphate kinase (PIPKIγ) via NF-κB signaling. Inhibition of exosomal PD-L1 secretion with the use of PIPKIγ or NF-κB inhibitor poses a potential strategy to overcome oxaliplatin resistance in gastric cancer [79]. Moreover, recent investigations shed new light regarding the influence of long-known drugs. For example, ibuprofen inhibits the secretion of PD-L1 in EVs [80].

Exosomes targeting the PD-1/PD-L1 signaling were studied as well. For example, Li et al. investigated the potential benefits of exosomes containing PD-L1 and CTLA-4 siRNA. SiRNA are small double stranded RNAs which bind to target mRNA leading to its degradation. Treatment with described exosomes in the xenograft colorectal cancer model resulted in reinvigoration of anti-tumor immune response and significant reduction in tumor growth [81,82]. Activated T-cells secrete exosomes exhibiting PD-1 on their surface. Exosomal PD-1 can bind to PD-L1 on cancer cells surface, which promotes its clathrin-mediated endocytosis. Moreover, exosomal PD-1 binds to exosomal PD-L1 and blocks its interaction with PD-1 on the T cells surface. Taken together, exosomal PD-1 promotes anti-tumor immune response by reducing PD-1/PD-L1 signaling. This was demonstrated to effectively reduce tumor growth in a mouse model of breast cancer [83].

### 2.2. Autoimmune Diseases

Autoimmune diseases represent a broad group of conditions with complex pathogenesis. Immunological components play a crucial role in the progression of diseases such as RA or SLE. Over the years, profound achievements have been made in the treatment of autoimmune diseases, as novel classes of drugs target cytokines and signaling pathways induced by abnormal inflammation present in the environment of affected tissue. By contrast to the pathophysiology of cancer, immunosuppression is a desired mechanism in autoimmunity. Immunotherapy in cancer patients can induce specific immune-related adverse events that are associated with autoimmunity.

The PD-1/PD-L1 axis is being investigated in the context of autoimmunity as well. However, it plays a diametrically different role than in cancer. As the interaction promotes immune tolerance, we aim to prevent the binding of receptor to its ligand in malignancies. By contrast, autoimmunity is associated with excessive immune system activation. Therefore, the promotion of PD-1/PD-L1 interaction and stimulation of immune tolerance in autoimmunity is desired.

To begin with, autoimmunity is frequently associated with an imbalance between lymphocyte subtypes. Imbalance between Th17 and Tregs presence is a frequent hallmark of autoimmune responses [84]. Moreover, this imbalance can accompany changes in PD-1 expression. For instance, Fang and collaborators examined peripheral T cells in patients with Hashimoto thyroiditis. The authors observed downregulation of PD-1 on Th17 cells from patients with thyroiditis compared to healthy controls [85]. The observed finding suggests that autoimmune Th17 cells can lose their regulatory abilities. Therefore, increasing the PD-1/PD-L1 binding or PD-1 expression could represent a beneficial mechanism in autoimmune diseases. This approach is utilized in the treatment with abatacept, a drug that mimics the activity of CTLA-4. Abatacept induces remissions and prevents the progression of RA [86,87].

PD-L1 of astrocytes can inhibit inflammatory reactions present in autoimmune neuroinflammation [88]. In addition, in a recent publication by Sugiura et al. [89], the authors described an interesting approach to enhance PD-1/PD-L1 signaling in autoimmunity. Specifically, the authors demonstrated that it is possible to suppress binding of PD-L1 with CD80 on antigen presenting cells, thus restoring the PD-1/PD-L1 activity. Similarly, mimics of PD-1L were also found to inhibit TCR signaling and suppress T cell activity [90].

EVs expressing PD-L1 were also examined in autoimmune conditions. As discussed previously, the properties of EVs frequently resemble those of their source cells. Mesenchymal stromal cells (MSCs) are immunoregulatory cells that demonstrated preclinical or clinical benefits in immune-mediated and autoimmune diseases, including psoriasis [33], ulcerative colitis [91], and RA [92], among many others. MSC-derived EVs can promote resolution of inflammation as well [93]. Xu et al. [94] combined the immunoregulatory roles of PD-L1 and MSCs-derived EVs through the development of MSCs-EV expressing PD-L1. In preclinical animal models, the authors proved that these structures can suppress autoimmune responses and alleviate inflammatory processes in ulcerative colitis and psoriasis. In psoriasis, researchers demonstrated that the use of MSCs-derived EVs expressing PD-L1 was associated with reduced presence of IL-17a+ and increased abundancy of FOXP3+ cells. Therefore, this treatment highlights the potential to reverse imbalances between Th17 and Treg cells present in autoimmunity. Additionally, EVs can be engineered to express PD-L1 [95].

**Figure 3:**
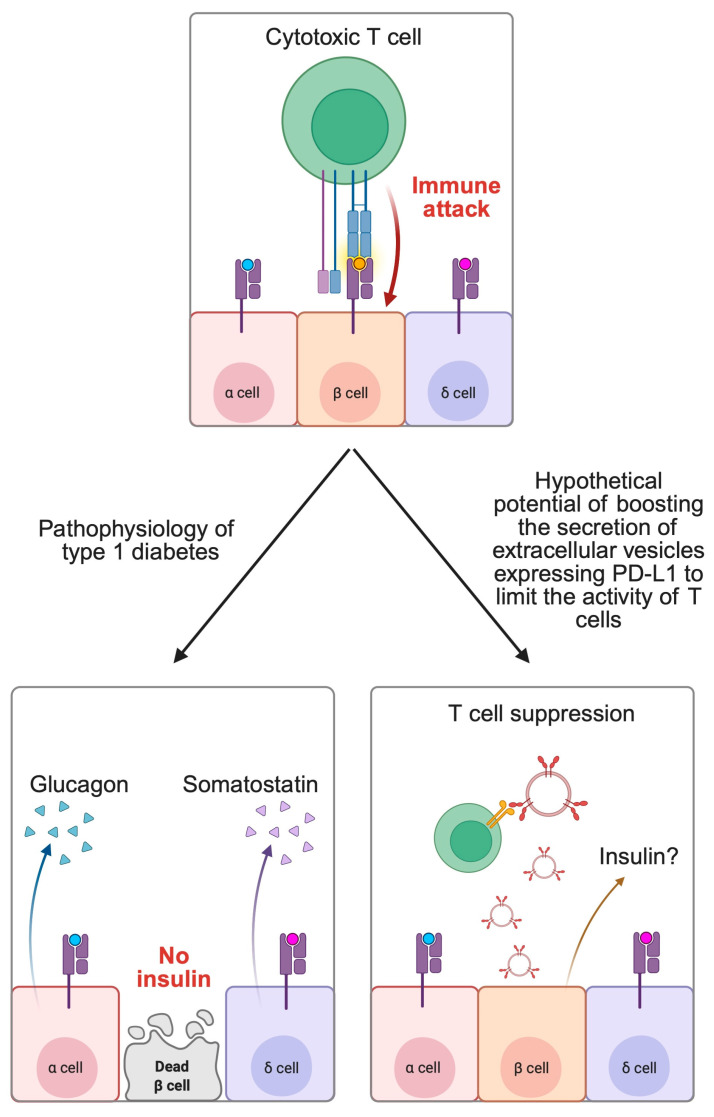
The potential role of extracellular vesicles expressing PD-L1 in the treatment of type 1 diabetes. Created in BioRender. Physiology, D. (2025) https://BioRender.com/0dl3sbw.

Type 1 diabetes (T1DM) is an autoimmune disorder associated with the presence of autoantibodies that damage the pancreatic beta cells, thus leading to insulin deficiency—the hallmark of T1DM. Recent studies demonstrated important relationships in PD-1/PD-L1 and the pathophysiology of T1DM. After exposure of IFN-α, beta cells can secrete EVs that express PD-L1. This property represents a crucial compensation feature of beta cells that try to suppress targeting and killing by the T cells. In murine models, EVs expressing PD-L1 were able to inhibit Tc cell proliferation. In clinical examinations, children with greater levels of circulating EVs-PD-L1 had increased concentrations of C-peptide, thus demonstrating residual pancreatic activity [94]. Hypothetically, this property could be further explored and developed to maintain the secretion of insulin in patients with T1DM. Such mechanism creates opportunities to reduce the risk of ketoacidosis or to implement therapeutic strategies that rely on residual pancreatic insulin secretion. Few recent studies suggest that this hypothesis could be true and potentially translate to clinical practice in the future. For example, the use of PD-L1 expressing platelets could reduce hyperglycemia and prevent the onset of T1DM in murine models [96]. Additionally, Yang and colleagues [97] evaluated the administration of engineered EVs expressing PD-L1 and Gal-9 in mice with diabetes. Researchers observed that the treatment could prevent the progression of T1DM and even restore normal blood glucose levels in approximately 60% of tested animals. Similar observations were noted in a recent work of Wang and collaborators [98] (Figure 3).

## 3. Conclusions

The present review demonstrates complex roles of PD-1 signaling in human diseases. In cancer, it is a crucial therapeutic target in the treatment of several malignancies. Monoclonal antibodies targeting PD-1 and PD-L1 entered daily clinical practice which is associated with improved outcomes. EVs with PD-L1 secreted by cancer cells contribute to cancer progression in several mechanisms. They suppress the anticancer activity of T cells and play a role in treatment resistance. Strategies that aim to suppress EV release by the cancer cells should be further investigated. They could potentially synergize with PD-1/PD-L1 targeting agents. By contrast, autoimmunity is associated with abnormal activity of the immune system, which targets its own cells and tissues. EVs containing PD-L1 represent a potential treatment strategy that could suppress inflammation and thus inhibit the disease progression. Current evidence demonstrates early stages of research utilizing EVs expressing PD-L1. Nevertheless, it should be mentioned that clinical translation of such studies will require a plethora of other studies. Furthermore, preclinical successes usually meet challenges when translating to clinical settings. Additionally, we must be careful when applying therapeutics modifying the immune checkpoints. Immunotherapy in cancer patients is associated with autoimmunity adverse events. It should be investigated whether PD-L1-based treatment strategies are associated with carcinogenesis in autoimmunity.

## Figures and Tables

**Table 1:** FDA approved monoclonal antibodies that belong to the cohort of immunotherapeutics. Based on search in FDA Label Database and List of Targeted Therapy Drugs Approved for Specific Types of Cancer (https://open.fda.gov/fdalabels/, accessed on 17 May 2025).

Drug Name	Mechanism of Action	Cancer Types	References
Avelumab	anti-PD-L1	Merkel cell carcinoma, Urothelial carcinoma, Renal cell carcinoma	[40]
Durvalumab	anti-PD-L1	Lung cancer (NSCLC and SCLC), Biliary tract cancer, Hepatocellular carcinoma, Endometrial cancer, bladder cancer	[41]
Atezolizumab	anti-PD-L1	Lung cancer (NSCLC and SCLC), Hepatocellular carcinoma, Melanoma, Alveolar soft part sarcoma	[42]
Nivolumab	anti-PD-1	Melanoma, NSCLC, Malignant pleural mesothelioma, Renal cell carcinoma, Classical Hodgkin lymphoma, HNSCC, Urothelial carcinoma, Colorectal cancer, Hepatocellular carcinoma, Esophageal carcinoma, Gastric cancer	[43]
Pembrolizumab	anti-PD-1	Melanoma, NSCLC, Malignant pleural mesothelioma, HNSCC, Classical Hodgkin lymphoma, Primary Mediastinal Large B-Cell Lymphoma, Urothelial Cancer, Microsatellite Instability-High or Mismatch Repair Deficient Cancer, Microsatellite Instability-High or Mismatch Repair Deficient Colorectal Cancer, Gastric Cancer, Esophageal Cancer, Cervical Cancer, Hepatocellular Carcinoma, Biliary Tract Cancer, MCC, RCC, Endometrial carcinoma, Tumor Mutational Burden-High (TMB-H) Cancer, Cutaneous Squamous Cell Carcinoma, Triple-Negative Breast Cancer	[44]
Dostarlimab	anti-PD-1	Endometrial Cancer, Mismatch Repair Deficient Recurrent or Advanced Solid Tumors	[45]
Retifanlimab	anti-PD-1	Merkel cell carcinoma	[46]
Toripalimab-tpzi	anti-PD-1	Nasopharyngeal carcinoma	[47]
Cemiplimab	anti-PD-1	Cutaneous squamous cell carcinoma, Basal cel cancer, NSCLC	[48]
Tislelizumab	anti-PD-1	Esophageal cancer, Gastric cancer	[49]

MCC—Merkel cell carcinoma, RCC—renal cell carcinoma, NSCLC—non-small cell lung cancer, SCLC—small cell lung cancer, HNSCC—head and neck squamous cell carcinoma,.

## Abbreviations

The following abbreviations are used in this manuscript:

EVs	Extracellular vesicles
MVBs	Multi-vesicular bodies
TME	Tumor microenvironment
DCs	Dendritic cells
PD-1	Programmed cell death protein 1
PD-L1	Programmed cell death protein ligand 1
ITIM	Immunoreceptor tyrosine-based inhibitor motif
ITSM	Immunoreceptor tyrosine-based switch motif
TCR	T cell receptor
BCR	B cell receptor
RA	Rheumatoid arthritis
SLE	Systemic lupus erythematosus
TAMs	Tumor associated macrophages
MDSCs	Myeloid-derived suppressor cells
CTLA-4	Cytotoxic T-lymphocyte-associated antigen 4
TIM-3	T cell immunoglobulin-3
TDEs	Tumor-derived exosomes
CPS	Combined positive score
ORR	Objective response rate
ICI	Immune checkpoint inhibitor
CTC	Circulating tumor cell
DLBCL	Diffuse large B cell lymphoma
NSCLC	Non-small cell lung carcinoma
MSCs	Mesenchymal stromal cells
T1DM	Type 1 diabetes melitus
